# Enhanced Osseointegration, Osteogenic Differentiation and Adherence Behaviour of Healthy Human Osteoblasts on a Roughened Titanium Surface by Vitamin K2 and Vitamin D3

**DOI:** 10.3390/ma18215012

**Published:** 2025-11-03

**Authors:** Katharina Tscheu, Ann Kathrin Bergmann, Christoph V. Suschek, Uwe Maus

**Affiliations:** 1 Clinic for Orthopaedics and Trauma Surgery, University Hospital of Düsseldorf, 40225 Düsseldorf, Germany; 2 Core Facility for Electron Microscopy, Faculty of Medicine, Heinrich Heine University Düsseldorf, 40225 Düsseldorf, Germany; kathrin.bergmann@uni-duesseldorf.de

**Keywords:** osseointegration, human osteoblasts, endoprosthesis, rough surface structure, vitamin K2, vitamin D3, adherence behaviour osteogenic differentiation

## Abstract

The number of endoprosthetic implants is constantly increasing. Successful osseointegration of the inserted material into the bone is essential for a prosthesis to remain in the bone as long as possible. In the clinical setting, a roughened titanium surface of implants is used as standard to enable the best possible osseointegration. Vitamin K2 and vitamin D3 play a decisive role in dynamic bone metabolism and therefore also influence osseointegration. For the first time, we carried out in vitro investigations with clinically relevant cells, primary healthy human osteoblasts (hOBs). We qualitatively compared the adhesion behaviour of hOBs on a plastic surface, a smooth, regular titanium surface structure and a roughened, irregular titanium surface structure by scanning electron microscopy and fluorescence microscopy. The osteogenic behaviour and the osteogenic differentiation capacity were quantitatively investigated by analysing the activity of alkaline phosphatase and the alizarin red S assay under the influence of vitamin K2, vitamin D3 and the combination of both vitamins. It was shown that more adhesion points formed between the cells and the titanium on the rough surface structure. In addition, a solid cell network developed more quickly on this side, with cell runners forming in three-dimensional space, which means the interactions between the cells across different cell layers. On the other hand, a structured cell network also appeared on the regular smooth surface structure, which means that the network seems to be formed and built up along a defined structure. The addition of vitamins further increased the osteogenic differentiation capacity on the rough titanium surface structure. In particular, the isolated addition of vitamin K2 showed an improved osteogenic differentiation in the long-term observation, whereas the combined addition of both vitamins promoted the initial osteogenic differentiation. Vitamin K2, therefore, plays a greater role in osseointegration than previously assumed. This opens up new possibilities for the use of vitamin K2 during and after the surgical insertion of an implant. The use of vitamin K2 should be reconsidered for clinical applications in implant care and further investigated clinically.

## 1. Introduction

The number of hip replacements performed in recent years has been steadily increasing [1]. Due to increasing life expectancy, it can be assumed that this trend will continue in the coming years [2,3]. How long an implanted endoprosthesis remains firmly in the bone depends largely on its osseointegration. On average, the lifespan of an implanted hip endoprosthesis is 15 years [4]. In recent years, numerous in vivo and in vitro studies have been carried out, which have looked at various aspects in order to improve osseointegration in the long term [5]. The term osseointegration means that the implants inserted are anchored directly in the bone. Furthermore, it means that the interaction between the implants and bone can only be cancelled by a fracture [6]. The understanding of osseointegration has significantly advanced arthroplasty, initially in dentistry, but ultimately also in orthopaedics [7].

Bone tissue is one of the most dynamic tissues in the body, so that even after a short time, bone metabolism adapts to new conditions [8]. Primary implant stability is crucial for implant integration in bone. The process of osseointegration takes place in three phases. In the first phase, the implant is inserted into the bone, and the bone cells make contact with the implant surface [9]. Here, it is crucial that the bone cells quickly become adherent to the newly inserted surface. Achieving a high level of adherence to the foreign material quickly is essential for the further course of osseointegration [10]. In the next phase, osteogenesis begins, which is primarily induced by inflammatory processes. In the third phase, remodelling introduced by angiogenesis and integration of the implant into the bone tissue begins [9]. However, the metabolic capacity and regenerative capacity of the bone do not play an essential role in the osseointegration process. Particularly in the first phase of osseointegration, the condition of the implant is crucial in order to initially optimise the conditions for firm cell adherence [11,12]. Research into improved osseointegration focuses, in particular, on the possibility of improving the surface quality of the implants inserted in order to improve the initial growth of bone cells [5].

Due to its biocompatibility, titanium has been the material of choice for the manufacture of endoprostheses for decades [13]. Titanium alloy forms an oxide layer with the surrounding air and is therefore particularly resistant to corrosion [14,15]. The titanium alloy TiAl6V4, in particular, is used as a standard, as it is considered to be highly biocompatible [13,16]. In addition to biocompatibility, the optimum implant surface must be such that the extracellular matrix (ECM) can form quickly [11]. How well the implant anchors in the bone depends finally on the interaction between the implant or its surface and the bone tissue and the surrounding environment [13]. Systematic studies have shown that a slightly roughened surface structure of the titanium promotes osseointegration, which is why this is now considered standard for implants [17]. 

The aim is to create an environment that promotes optimal regeneration of bone tissue after the insertion of a titanium implant. The modification of the surface represents a research approach with regard to a possible longer service life of implants [18].

The overall aim is to permanently extend the half-life of endoprostheses and strengthen the implant–bone interaction [19].

Due to increasing life expectancy, it can be assumed that the number of hip arthroplasty revisions will increase further in the coming years, too [20]. At the same time, the bone quality that is encountered will deteriorate, and the need for implantation of an endoprosthesis will increase even more [21]. This makes it all the more important to understand how osseointegration works in healthy bone and how it can be continuously improved, in order to then determine how this can also be achieved in weakened bone tissue [22]. The clinical aim must therefore be to ensure that an implanted endoprosthesis remains firmly anchored in the bone for as long as possible. Moreover, no reoperation or reimplantation should be necessary. For this, it is crucial to understand the exact processes of healthy human osteoblasts in interaction with the rough implant surface.

This study investigates another aspect that can extend the service life and improve the anchoring of the implant in the bone. It is known from numerous studies that vitamin D3 and vitamin K2 play a decisive role in bone metabolism and favour bone growth [23].

Vitamin D is a crucial building block in this process. The presence of vitamin D and its metabolites promotes cell growth and increases the formation of fibronectin [24]. The active form of vitamin D is vitamin D3. The formation of the ECM and thus bone mineralisation is significantly controlled by vitamin D3 [25]. This takes place via direct and indirect processes. Vitamin D3 binds directly to the vitamin D3 receptor of osteoblasts and thus activates mineralisation [26]. Vitamin D3 indirectly regulates bone mineralisation by controlling the calcium and phosphate balance [27]. Vitamin D supplements, in particular, have been used for decades to prevent osteoporosis [28]. An adequate supply of vitamin D3 can significantly delay and reduce the risk of the development of osteoporosis. This is also associated with a reduced risk of fractures [29,30]. There is clear evidence that vitamin D deficiency in animal models has a negative effect on the osseointegration of an implant [31].

However, the actual influence of vitamin K2 on the proliferation and differentiation of osteogenic differentiating cells has not yet been conclusively determined. So far, vitamins K1 and K2 are mainly known for their influence on haemostasis as cofactors for the formation of vitamin K-dependent coagulation factors [32]. However, it is now also known that vitamin K2 has a relevant influence on bone balance [33]. However, there are indications that vitamin K2 has a positive effect on the effect of vitamin D3 and the deposition of osteocalcin in the extracellular matrix (and the associated increase in differentiation) [34]. Vitamin K2 also inhibits the activity of osteoclasts and, at the same time, promotes the transformation of osteoblasts into osteocytes [35,36,37]. Overall, vitamin K2 appears to be involved in many metabolic processes of bone. However, unlike vitamin D3, it is not yet used as a standard for the prevention of osteoporosis; only in Japan has vitamin K2 been part of the treatment of osteoporosis for many years [38].

For the first time, the processes of osteointegration and adherence behaviour are investigated using clinically relevant cells in primarily healthy human osteoblasts. Furthermore, this work now brings together the various aspects of promoting osseointegration. In addition to the detailed presentation of how vitamin D3 and vitamin K2 promote the formation of complex cell clusters, the influence of a roughened surface structure on the differentiation capacity of human osteoblasts is also investigated.

## 2. Materials and Methods

### 2.1. Titanium Preparations

The used titanium samples (provided by Peter Brehm GmbH, Weisendorf, Germany, for this in vitro analysis) had a diameter of 14.7 mm and a thickness of 2.0 mm. With this size, they fit exactly into one well of a 24-well plate. They were made of the commercially available titanium alloy TiAl6V4 and had a roughened (Rz value of 40–60 µm) and a smooth polished side (Rz value approx. 10 µm). The Rz value refers to the maximum difference between a peak and a valley on the surface. The smooth side is normally not foreseen for direct contact with bone and thus served as the control. As a limited number of samples were used, it was necessary to process the plates after use. Therefore, a cleaning protocol was established for the reuse of the titanium preparations in which the surface structure was completely preserved, but the biological cell material was removed. Therefore, the titanium discs were soaked in acetic acid (Sigma-Aldrich Co., St. Louis, MO, USA) for 60 min and washed overnight in distilled water. The titanium discs were then cleaned mechanically for 15 min using ultrasound and finally autoclaved at 128 °C.

### 2.2. Cells

Human osteoblasts (hOBs) were isolated from femoral heads after removal due to a finding of arthrosis or after a fracture event. The procedure was authorised by the Research Ethics Committee of the Heinrich Heine University Düsseldorf (Study No. 5585R). The patients consented in writing to the further use of the removed cells. The patients from whom the cells were taken were male and female, aged between 24 and 86 years. In a previously performed Dual-Energy X-ray absorptiometry measurement (DXA) of the spine and proximal femur, the hOBs were defined as T > −2.5. This means that the bone from which the cells were extracted was healthy and non-osteoporotic [39]. All measurements and the removal of the femoral heads were medically indicated, and all included persons consented to the further use of the removed tissue. All surgical tissue removals were performed at the Department of Orthopaedics and Trauma Surgery, University Hospital Düsseldorf. Femoral heads were transferred immediately to the lab or were stored at 4 °C upon further processing. 

Firstly, the spongiosa was separated from the compacta using a sharpened spoon. The resulting fragments were transferred to a tube. Into this tube, 0.25% collagenase type IV (Life Technologies Ltd., Thermo Fisher Scientific, Waltham, MA, USA) in wash medium (1% penicillin/streptomycin 10,000 U/mL/10 mg/mL in GibcoTM Ham’s F-12 Nutrient mix from Life Technologies Ltd., Thermo Fisher Scientific, Waltham, MA, USA) was filtered in sterile conditions. Everything was incubated for 2 1/2 h at 37 °C with continuous shaking. The solution was then carefully transferred to a new tube, which was centrifuged for 5 min at 400× *g*. The centrifuged supernatant was removed, and the resulting pellet was resuspended in the wash medium. Again, the solution was centrifuged for 5 min at 400× *g*. The supernatant was removed again. Afterwards, the pellet was resuspended with standard cell medium and plated in T75 cell flasks [40,41].

The hOBs were cultured in standard cell culture medium (DMEM, (Life Technologies Ltd., Thermo Fisher Scientific, Waltham, MA, USA) to which 5% Hepes (Sigma-Aldrich Co., St. Louis, MO, USA), 5% penicillin/streptomycin (P/S; PAN Biotech, Aidenbach, Germany) and 10% foetal bovine serum (FBS; Sigma-Aldrich Co., St. Louis, MO, USA) were added. The incubation was carried out at 37 °C, 5% CO_2_ and 100% humidity in the dark.

The grown hOBs were regularly monitored under a light microscope. Their growth and vitality were documented. The cell culture medium was changed at least twice a week to optimise growth and prevent possible contamination. If a continuous, uniform cell lawn was detected with the light microscope, the cells were split into several cell culture flasks. To accomplish this, the cell culture medium was first removed from the cell culture flask, and the hOB was washed with Dulbecco’s Phosphate-Buffered Saline, modified, without calcium, chloride and magnesium (PBS; Sigma-Aldrich Co., St. Louis, MO, USA). To mobilise the hOBs, a solution of PBS with 10% trypsin (Sigma-Aldrich Co., St. Louis, MO, USA) was added to the cells. These were incubated for 5 min at 37 °C, 5% CO_2_ and 100% humidity in the dark. Under the light microscope, we checked whether the cells had successfully detached from the cell culture flap. Any hOBs that had not yet been detached were carefully removed from the plastic surface of the cell culture flask using a soft cell culture scraper. To inhibit further mobilisation of the cells, the same amount of cell culture medium was added as the previously added trypsin–PBS solution. This solution was transferred to a tube and centrifuged for 5 min at 300× *g*. The supernatant was removed, and the cell pellet was resuspended in the cell culture medium. Then, the number of hOBs could be determined using a Neubauer counting chamber under a light microscope, or they could be distributed evenly directly to new cell culture flasks.

### 2.3. Cultivating and Differentiation During the Experiments

For the experiments, the cells were removed from the cell culture flasks as described and transferred to 24-well plates or to the titanium plates contained therein. The control group always consisted of hOBs on the plastic surface in the well plate, which, in contrast to the cells on the titanium plates, could still be monitored by light microscopy. Per well, 20,000 hOBs were always initially plated in 1 mL of cell culture medium. This meant that the titanium plates were also well covered with cell medium. After 72 h of growth, the cell culture medium was carefully removed and replaced by a differentiation medium, which also contained FBS and P/S in the same amount as the standard medium and additionally contained Dexamethason (Sigma-Aldrich Co., St. Louis, MO, USA), L-Ascorbin-2-Phosphat (Sigma-Aldrich Co., St. Louis, MO, USA) and ß-Glycerophosphat (Sigma-Aldrich Co., St. Louis, MO, USA) [42]. If differentiation was also to be carried out with the addition of vitamins, vitamin K2 (concentration of 10 µM; Sigma-Aldrich Co., St. Louis, MO, USA), vitamin D3 (concentration of 10 nM; Sigma-Aldrich Co., St. Louis, MO, USA) or a combination of both was added to the differentiation medium from the first addition. The differentiation medium with and without vitamin supplements was renewed every 48 h. The results were analysed after 1, 7, 14 and 21 days of differentiation. Two discs with the same treatment, but without cells, served as blanks.

### 2.4. Quantitative Analysis of the Differentiation Capacity and Development of the Extracellular Matrix (ECM)

#### 2.4.1. Alkaline Phosphatase (ALP)

The enzymatic activity of ALP in the cells was used as an osteogenic differentiation marker. At the end of the defined differentiation time, the cells or platelets with cells were first washed with PBS and then incubated with 4-nitrophenol solution (Sigma-Aldrich Co., St. Louis, MO, USA) for 20 min in the dark at room temperature. The solution was transferred to 96-well plates; duplicates were always prepared. The mean value of the empty titanium plates carried served as a blank and was subtracted from the values obtained for the plates with hOBs. The discoloured supernatant was measured at 405 nm using a microplate reader [43].

#### 2.4.2. Alizarin Red S Staining

Staining with Alizarin Red S served as a later osteogenic marker of the expression of the ECM. At the end of the defined differentiation time, the samples were washed with PBS and fixed with ROTI^®^ Histofix (Carl Roth GmbH + Co. KG, Karlsruhe, Germany) for 15 min at 37 °C. The fixed cells on the platelets and in the wells were washed with distilled water and incubated with 0.5% Alizarin Red S monosodium salt in distilled water for 20 min at 37 °C. Calcium is deposited in differentiated cells and forms complexes with the alizarin dye [44]. Due to the previous fixation, only excess dye was subsequently removed. After washing three times with distilled water, the cells were redissolved using a 10% cetylpyridinium chloride solution (Sigma-Aldrich Co., St. Louis, MO, USA) for 60 min on a small plate shaker (type KM2, Edmund Bühler GmbH, Bodelshausen, Germany) at 250 rpm. This made it possible to ensure gentle removal even from the rough side of the discs without having to mechanically mainpulse the titanium surfaces. The concentration of the redissolved staining was measured at 450 nm in the microplate reader. The mean value determined from the bare titanium plates was subtracted from the values of the differentiated hOBs on the titanium surfaces.

### 2.5. Visualisation of Cell Development

The first qualitative evaluations were carried out after 48 h of growth to visualise the basic cell adherence. No differentiation took place at this stage; the cells simply grew on the titanium preparations without further treatment.

#### 2.5.1. Scanning Electron Microscope (SEM)

Scanning electron micrographs were taken on both sides of the platelets without and with hOBs to visualise cell distribution and cell adherence. For a detailed examination of the surface properties of the platelets, the platelets without grown cells were analysed under a scanning electron microscope. Both new platelets that had never been used before and native platelets that had already been used for the experiments were visualised. These platelets were dried over several days at 37 °C, 5% CO_2_ and 100% humidity in the dark.

To visualise the platelets with cells, the samples were first washed with PBS and then fixed with fixative containing 4% paraformaldehyde (Merck KGaA, Darmstadt, Germany) and 2.5% glutaraldehyde (Sigma-Aldrich Co., St. Louis, MO, USA) in 0.1 M cacodylate buffer (Sigma-Aldrich Co., St. Louis, MO, USA) overnight at 5 °C. The fixed cells were washed three times with distilled water for 10 min on the plate shaker and then dehydrated with ascending concentrations of acetone, starting with 30% acetone (Merck KGaA, Darmstadt, Germany) in distilled water. The platelets with 70% acetone were prepared at 5 °C overnight. After dehydration with 100% acetone, critical point drying was performed. The titanium plates with hOBs were sputter-coated with a 20–24 nm thick layer of gold (108 auto, Cressington Scientific Instruments, Watford, UK). The recording points of the first comparison discs (new discs and discs with newly grown cells) were selected at random, while comparable geographical points on the respective discs were selected for subsequent images. All images were taken using a Focused Ion Beam Scanning Electron Microscope (FIB-SEM) (Crossbeam 550, Carl Zeiss AG, Oberkochen, Germany).

#### 2.5.2. Visualisation by Immunofluorescence

##### Calcein

The visualisation of the developed ECM in the cells by calcein staining served to qualitatively illustrate the development of the degree of calcification. Calcein binds to calcium and calcium carbonate deposits in the extracellular matrix [2,3,45]. For this purpose, the cells were first washed with PBS and then stained with a solution of calcein (ApolloScientific Ltd., Stockport, UK) in PBS for 20 min at room temperature in the dark. After washing three times with PBS, Hoechst (ThermoFisher Scientific Ltd., Waltham, MA, USA) was added to the cells. After another 5 min incubation, the cells were washed again with PBS, and the stained samples were observed under a fluorescence microscope (Axiovert 200; Carl Zeiss AG, Jena, Germany). Once an overview of the cell distribution was obtained, general and detailed photographs were taken.

##### Vinculin

Staining with vinculin and phalloidin was used to visualise the cell cytoskeleton, punctual cell–cell and cell–titanium contacts. Additional staining of the cell nuclei with 4′,6-Diamidin-2-phenylindol (DAPI) was used to visualise the cell distribution and to better distinguish individual cells. For this purpose, the instructions from Sigma-Aldrich Co., St. Louis, MO, USA, were used. The cells on the titanium plates and in the well plates were first washed with PBS and then fixed with 4% paraformaldehyde (Merck KGaA, Darmstadt, Germany) for 20 min at room temperature. Then, the cells were first washed with PBS once and then washed with wash buffer containing 0.05% Tween-20 (Sigma-Aldrich Co., St. Louis, MO, USA) in PBS. The cells were permeabilised with 0.01% Triton (Sigma-Aldrich Co., St. Louis, MO, USA) for 5 min at room temperature. The cells were washed again with the wash buffer, and a block solution containing 1% bovine serum albumin (Sigma-Aldrich Co., St. Louis, MO, USA) was added for 30 min at room temperature. The blocking solution was removed, and the vinculin antibody (V9131, Sigma-Aldrich Co., St. Louis, MO, USA) was added at a ratio of 1:400. The cells were incubated for 1 h at room temperature. The cells were then washed three times for ten minutes with the wash buffer, and the second antibody (Goat anti-Mouse IgG (H + L) Cross-Adsorbed Secondary Antibody; ThermoFisher Scientific Ltd., Waltham, MA, USA) and phalloidin (ThermoFisher Scientific Ltd., Waltham, MA, USA) were added to the cells for 1 h. After washing three times with wash buffer, 4′,6-diamidino-2-phenylindole (DAPI; ThermoFisher Scientific, Ltd., Waltham, MA, USA) was finally added for 5 min, and the platelets and well plates were observed under a fluorescence microscope (Axiovert 200; Carl Zeiss AG, Jena, Germany). An overview of the cell distribution was first obtained at low magnification. The recording points were then selected at random, with a particular focus on detailed images of cell–titanium contacts.

### 2.6. Statistical Analysis

Graph Pad Prism software version 10.3 (Boston, MA, USA) was used for the statistical analysis. In order to eliminate the first-order error at a level of α = 0.05, a significance level of 5% was selected (*p* < 0.05). The significance level is marked with an asterisk, where two asterisks indicate a level of *p* < 0.01 and three asterisks stand for *p* < 0.001. Instances of no significant differences are not marked separately. All quantitative results are presented as box plots or as bars, in which the mean value and the standard deviations are shown. The *t*-test and the Mann–Whitney U-test were used for the statistical comparison of two groups. 

The quantitative analysis of the recorded fluorescence images with vinculin and phalloidin was performed with the programme ImageJ (Version 1.53m; Wayne Rasband and contributors, National Institutes of Health, Bethesda, MD, USA). All images analysed were taken on the same day using the same staining protocol and the same microscope with the same settings. ImageJ was first used to isolate the green colour signal and then to calculate the number of pixels with this colour signal.

## 3. Results

### 3.1. Visualisation of Cell Adherence and Cell Differentiation

#### 3.1.1. SEM

**Figure 1:**
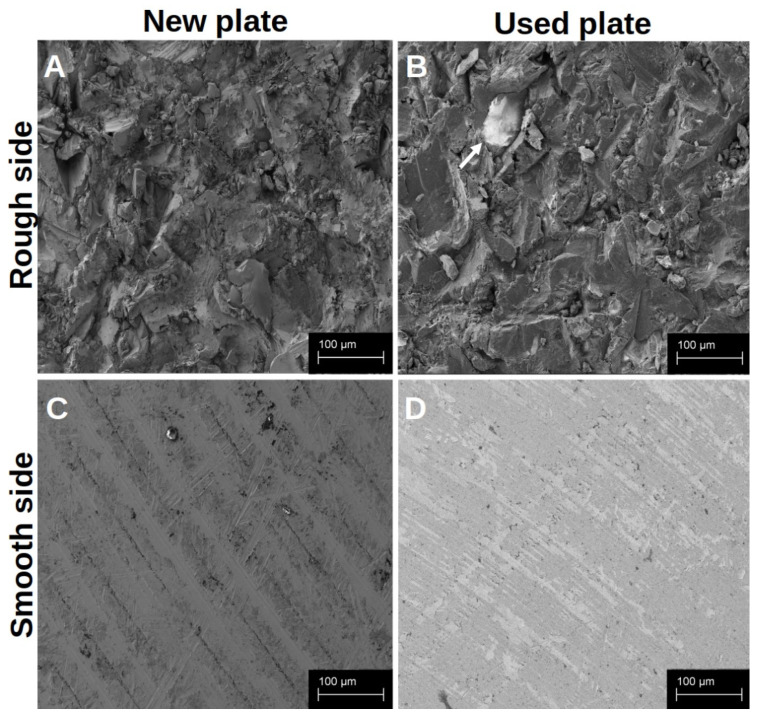
Visualisation of the different surface structures of the native platelets in the SEM. The platelets were analysed immediately after receipt and unused (**A**,**C**); after they had already been used, the platelets were dried for 7 d and then examined in the SEM without further treatment (**B**,**D**). The arrow marks a small residue of organic material on the rough surface structure.

Both sides of the native titanium platelets could be visualised in the SEM (Figure 1). The smooth side of each platelet showed a regular structure arranged in even rings around the centre of the platelet, with only slight elevations and unevenness (Figure 1C,D). The centre of the platelet, on the other hand, appeared somewhat irregular. The rough side of the disc, on the other hand, was very irregular. There was a pronounced three-dimensional structure with some sharp and smooth edges (Figure 1A,B).

In order to detect differences between the new and reused discs at an early stage and to determine whether the established cleaning protocol works, we examined them under a scanning electron microscope. When comparing the new platelets with the previously used platelets (at least 20 reuses before), it was noticeable that the previously used preparations showed slight signs of wear, but hardly any organic material remained after cleaning. The basic characteristics of the structures on both sides of the discs were retained. The traces of use were more noticeable on the smooth side (Figure 1D) than on the rough side, whereas biological material was more likely to remain on the edges that were difficult to see, particularly on the rough structure (Figure 1B).

**Figure 2:**
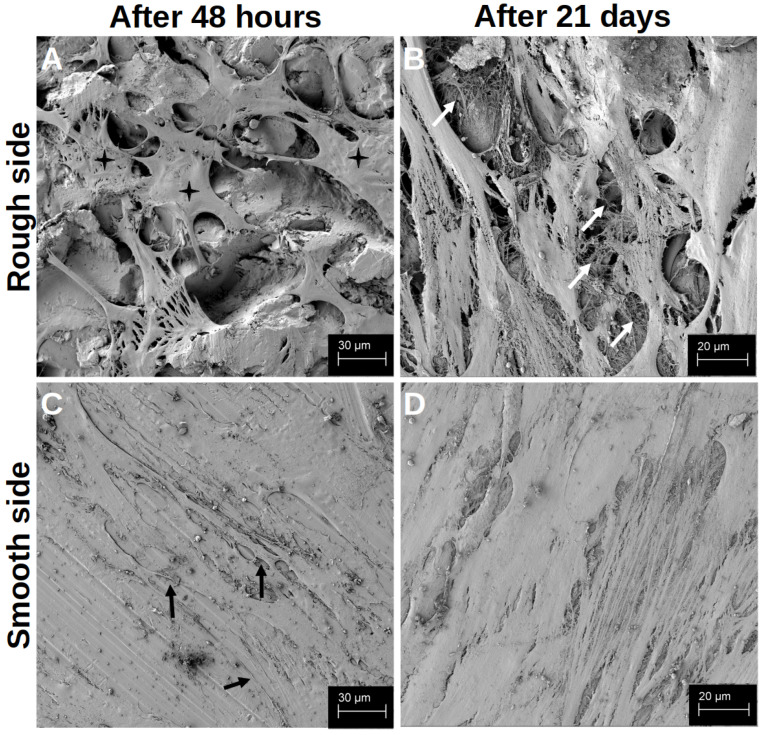
Visualisation of the differentiation after 48 h (**A**,**C**) and after 21 d (**B**,**D**) on the rough side of the platelets (**A**,**B**) and on the smooth side (**C**,**D**) in the SEM. The black crosses in (**A**) mark the space-filling cell bodies. The white arrows in (**B**) highlight a few cross-links between the cells and the cross-links between the cells and the material. The black arrows in (**C**) mark a few cell–cell junctions that cross the structured surface.

**Figure 3:**
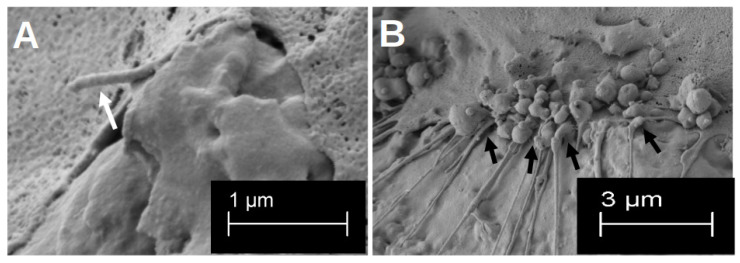
Visualisation in the SEM of the three-dimensional formation of podocytes after 48 h of growth (**A**). The white arrow highlights one clear three-dimensional podocyte. Visualisation of the multiple formation of cell–titanium contacts after 14 d of differentiation on the rough surface structure (**B**). The cell–material interactions are marked by the black arrows.

After the initial growth phase of 48 h, hOBs appeared on both sides of the platelets, with a regular and structured arrangement on the smooth side (Figure 2C). The cells accumulated along the slightly predetermined structure, but they also formed cell–cell junctions that crossed these structures (black arrows in Figure 2C). In addition, the hOBs morphologically appeared as flat and planar cells. Occasionally, morphologically different cells were recognisable. On the rough side, a three-dimensional growth with pronounced podocytes and cell–cell interactions was observed (white arrows in Figure 2A). In this context, the term ‘podocytes’ refers to the formation of long-distance cell extensions with which the cells interact with other cells and the material. The cells took on the uneven structures of this side and enclosed entire titanium formations. In particular, the formation of podocytes in the space could be observed (Figure 3A). While the cell–cell interactions on the smooth side were created by clearly defined podocytes in the plane, the podocytes on the rough side developed in all directions. Overall, there were more cell–cell interactions on the rough side, which appeared more variable in shape but often also more fragile. Even after 14 d of differentiation, multiple anchorages and cell–titanium contacts could be observed on the raised structures (white arrows Figure 3B). The flat morphology of the cells was also reflected in the rough surface structure, with the cell bodies filling the spaces between the raised structures (black crosses in Figure 2A).

After 21 d of differentiation, cell differentiation was so pronounced that no titanium surface was recognisable in the SEM image on either side of the preparation (Figure 2B,D). On the contrary, several cell layers could be identified on top of each other on both sides of the preparation, with at least six layers on top of each other on the rough side. Here, a distinct network was found that connected the cells with each other in a variable manner, so that the cross-linking of the cells also extended beyond one cell layer (white arrows in Figure 2B). On the smooth side, the formation of the network was less variable, but rather structured and uniform (Figure 2D).

#### 3.1.2. Immunofluorescence

##### Vinculin and Phalloidin

**Figure 4:**
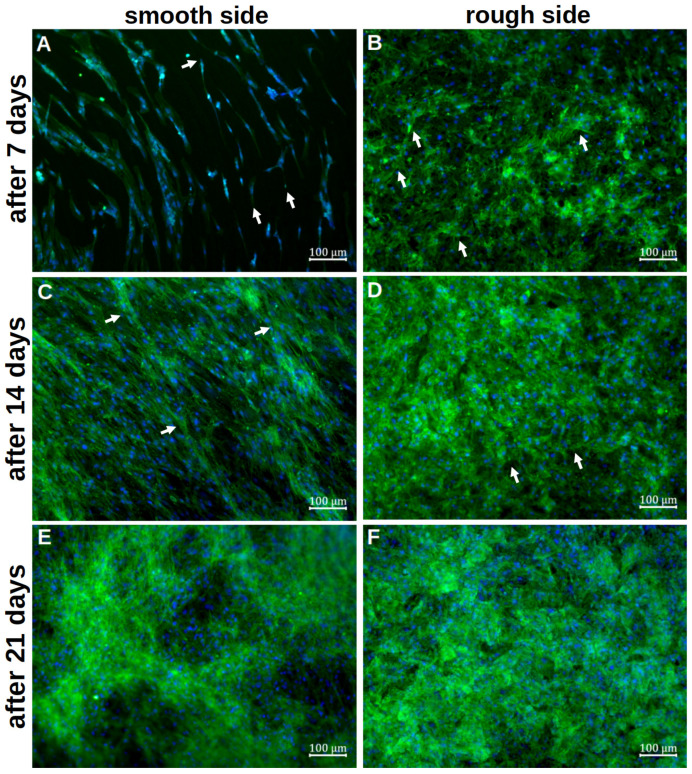
Representative visualisation of the cell adherence and contribution on the smooth titanium surface (**A**,**C**,**E**) and on the rough titanium surface (**B**,**D**,**F**) after 7 days (**A**,**B**), 14 days (**C**,**D**) and 21 days (**E**,**F**) of differentiation by using vinculin, phalloidin and DAPI staining. The white arrows in (**A**,**B**,**D**) mark exemplary cell–titanium interactions, whereas the white arrows in (**C**) highlight a few cells that cross the ring structure.

Staining with vinculin and phalloidin revealed clear cell–cell contacts and cell–platelet interactions. On the smooth side, the cells initially appeared organised and flat after 7d of differentiation (Figure 4A). The basic structure of the cytoskeleton followed the rings already seen in the SEM. No planar contact between the cells and the metal could be identified. Cell–titanium interactions were seen selectively and at the cell extensions. The first cells grouped together, but individual cells without further cell contact could also be identified. After only 14d, the titanium base could no longer be recognised, as a densely woven cell network consisting of several cell layers appeared (Figure 4C). The basic order of the initial deposition could still be partially recognised, with numerous transverse cells and cells crossing the ring structure. There were also numerous cell–cell interactions, whereas cell–titanium contacts were not evident. After 21 d of differentiation, this network appeared again (Figure 4E). Qualitatively, it was no longer possible to determine how it changed during further differentiation.

On the rough side, an interwoven network of cells was already visible after the initial growth phase (Figure 4B). A basic structure or system could not be discerned here. Several layers of cells could already be visualised at this early stage. Overall, there were more cell–titanium contacts than on the smooth side. Multiple cell–preparation interactions could be observed, particularly on flat, inclined surface elements. Individual cells were no longer recognisable. After a further 7 d of differentiation, the formed network appeared even denser in most places, so it was not possible to determine how many cell layers it consisted of (Figure 4D). In some places, even deeper layers of the network could be identified, where cell–titanium contacts could still be seen. After 21 d, the cell network was so pronounced that it was no longer possible to identify individual cells (Figure 4F). Only in a few places could the cytoskeleton of individual cells still be clearly identified. An orientation of the cells was not recognisable.

**Figure 5:**
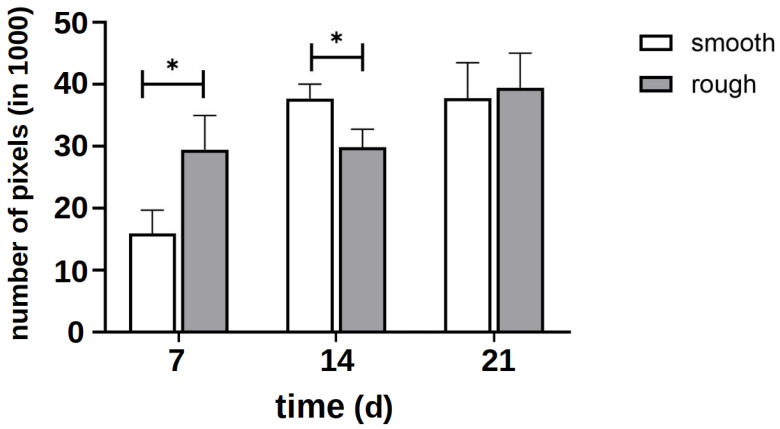
Quantitative evaluation of the intensity of the green signal of staining with vinculin and phalloidin on several representative images of the smooth and rough side after 7 days of differentiation without the influence of vitamins. The asterisk marks the significance level of *p* < 0.05.

The quantitative evaluation of the intensity of the green signal (Figure 5) showed that after 7 d, it formed more strongly on the rough side; thus, initially, more cytoplasm and cytoskeleton formed on this side. After 14 d, however, it became apparent that a clear cell network had also formed on the smooth side. After 21 d, there was no difference between the two sides. Due to the clinical relevance of the rough side, further investigations focused on this side.

**Figure 6:**
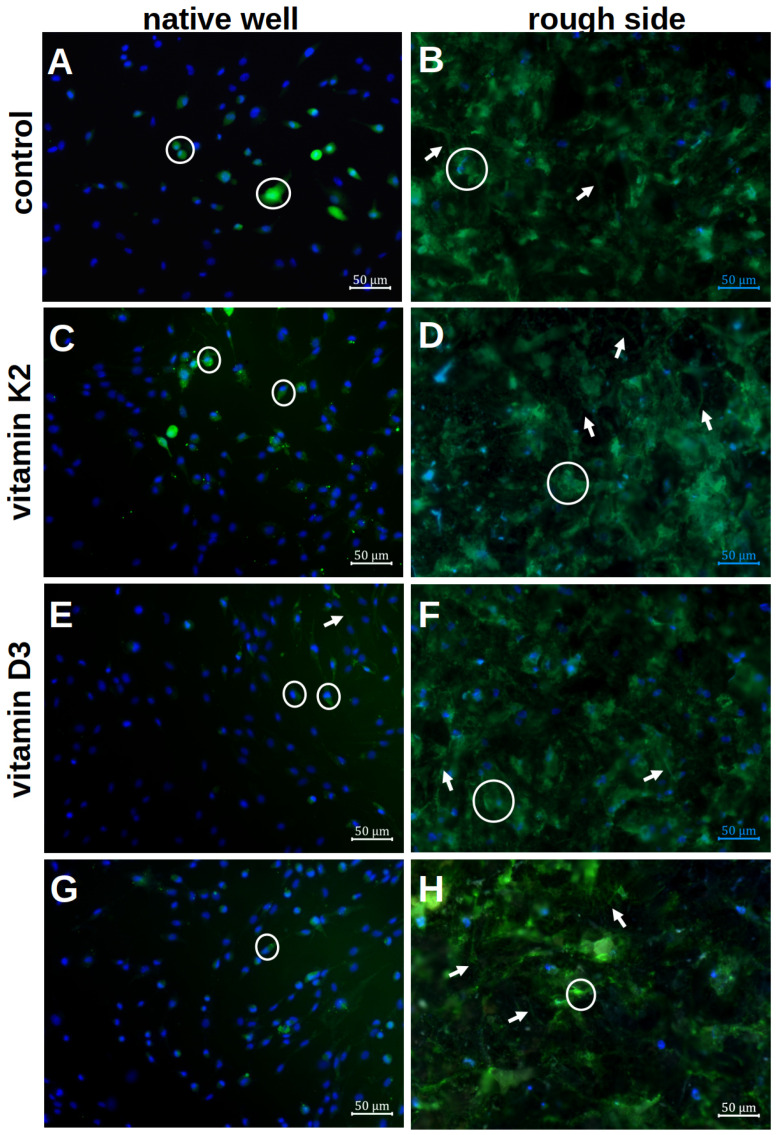
Visualisation of the mineralisation of hOBs after 7 d of differentiation in the native well (**A**,**C**,**E**,**G**) and on the rough titanium surface (**B**,**D**,**F**,**H**) with the different vitamin treatments by using calcein staining and Hoechst. The white rings highlight exemplary cells with an ECM halo, whereas the white arrows stress the podocytes.

The results of the calcein staining are shown in Figure 6.

The formation of the ECM on the rough platelet side and in the well with and without the influence of vitamin additions was qualitatively analysed after 7 d of differentiation. In the native well, only small ECM halos could be visualised directly around the cell nuclei (white rings in Figure 6A). There were only isolated cell–cell contacts that were formed via the ECM. When vitamins were added, more podocytes and larger ECM areas were visible. The clearest podocytes were seen when vitamin K2 was administered alone (white arrows in Figure 6C). With the addition of vitamin D3, however, more cell–cell contacts could be visualised (Figure 6E). A combination of both effects was not seen when both vitamins were added together (Figure 6G).

By staining the rough surface structure with calcein, more calcium could be stained qualitatively in the ECM. Even without the addition of vitamins, the ECM was more distributed than in the well (Figure 6B). A clear distribution around the cell nuclei could not be visualised. When vitamin K2 was added, there was no clear change compared to the native visualisation (Figure 6D). However, more stained ECM was visible under vitamin D3 (Figure 6F). In addition, developed podocytes could be visualised, so that a clear network had formed on the rough surface structure. This was also visible when both vitamins were added, although fewer cell nuclei could be visualised (Figure 6H). However, a combination of a delicate network and a flat ECM formation could again be observed.

### 3.2. Differentiation and Mineralisation

#### 3.2.1. ALP (Figure 7)

In the initial phase of differentiation after 24 h, the ALP activity on the rough surface structure of the titanium preparations was significantly more pronounced than in the native well. The addition of vitamin K2 or vitamin D3 alone did not support this effect. However, there was a significantly higher ALP activity under the influence of vitamin D3 than under the influence of vitamin K2. The combined administration of both vitamins was significantly superior to both vitamin additions alone. This showed the most pronounced ALP activity and thus differentiation. This picture changed after 7 d of differentiation. Here, the hOB on the rough side with the addition of vitamin K2 showed the highest ALP activity, which was significantly higher than that of the untreated titanium preparations, the administration of vitamin D3 alone and the combined administration. Even after 14 d, the ALP activity was significantly higher with the vitamin K2 administration than with the vitamin D3 administration. In contrast, the highest ALP activity was observed with the combined vitamin administration. This was significantly superior to all other treatments at this point. 

**Figure 7:**
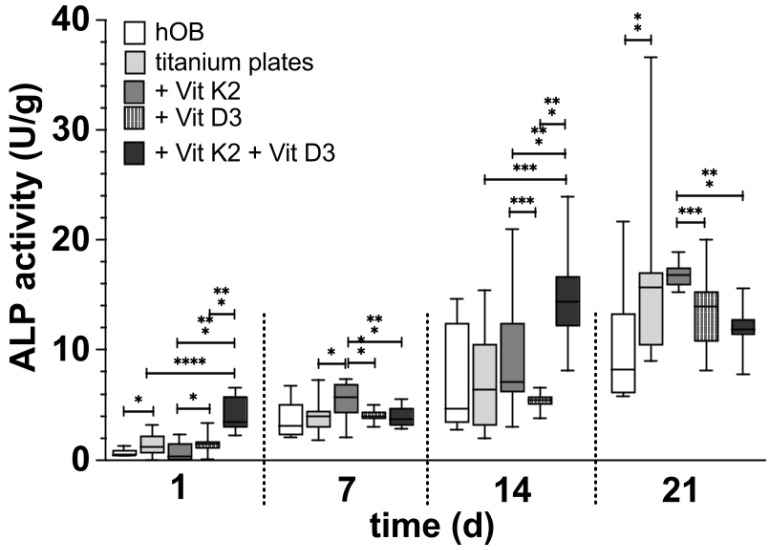
The development of ALP activity in hOBs over the time in the native well (white), on the rough titanium surface without vitamins (light grey), on the rough titanium surface under the influence of vitamin K2 (grey), on the rough under the influence of vitamin D3 (black with white spots) and on the rough titanium surface under the influence of both vitamins (black). The asterisks indicate the different significance levels. One marks *p* < 0.05, two marks *p* < 0.01 and three stand for *p* < 0.001 and four for *p* < 0.0001.

In the long-term observation after 21 d of differentiation, the ALP activity on the native titanium platelets already appeared significantly superior to the native cells in the well. An increase in this effect by vitamin addition could not be observed. ALP activity with the addition of vitamin K2 appeared to be at a similar level to the nontreated preparations. On the other hand, ALP activity and thus differentiation were significantly increased compared to the treatment with vitamin D3 and combined vitamin administration.

#### 3.2.2. Alizarin Red S Staining (Figure 8)

After just one day of differentiation, the rough surface structure without the addition of vitamins showed a significantly more pronounced mineralisation of the hOBs compared to those that differentiated in the native well. Both the individual addition of vitamins was superior to differentiation on the native titanium surface structure and the combined addition of both vitamins. This was also significantly superior to the two individual additions of the vitamins. After 7 d of differentiation, the degree of mineralisation of the hOBs was significantly higher under all treatments on the titanium preparations than in the native well. A significant difference between the different vitamin additions could not be identified. After 14 d of differentiation, the degree of mineralisation was at a similar level in all the samples. The degree of mineralisation in the native well was also similar compared to the previous tests. Only the treatment with both vitamins on the rough side proved to be significantly different from all treatments in terms of mineralisation. In the long-term observation after 21 d, it was noticeable that a similar level of mineralisation developed between the native hOBs in the well and the native hOBs on the rough surface structure. The treatment with the different vitamins was significantly higher than the native hOBs on the rough side, whereby the hOBs treated with vitamin D3 were significantly less mineralised on the rough side than with vitamin K2 and the combination of both vitamins. There was no significant difference between the treatment with vitamin K2 and the vitamin combination.

**Figure 8:**
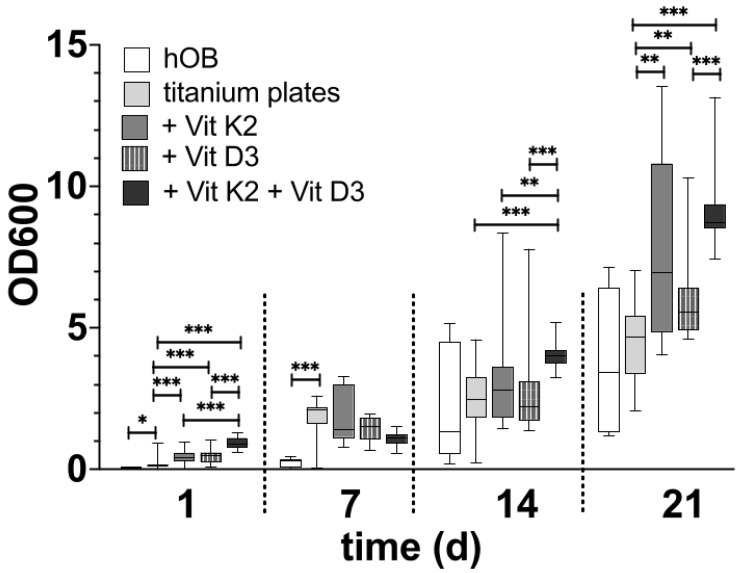
The development of the grade of mineralisation in hOBs over the time in the native well (white), on the rough titanium surface without vitamins (light grey), on the rough titanium surface under the influence of vitamin K2 (grey), on the rough under the influence of vitamin D3 (black with white spots) and on the rough titanium surface under the influence of both vitamins (black). The significance levels are marked by the asterisks. The significance level of *p* < 0.05 is marked with one asterisks, two stand for *p* < 0.01 and three mark *p* < 0.001.

## 4. Discussion

### 4.1. Role of Titanium in the Process of Osseointegration

The differentiation (Figure 7) and mineralisation (Figure 8) of the hOBs on the titanium platelets were significantly faster than in the native well, especially initially. Since this development could be seen in the fluorescence image and the SEM on both sides of the platelets, it can be assumed that the material itself, TiAl6V4, has a positive effect on the course of these processes. When a hOB comes into contact with titanium, there appears to be an increased release of cytokines, which ensures the rapid progression of differentiation [46,47]. This is essential for successful osseointegration [22]. More and more, it has to be said that osseointegration is an inflammatory process [48]. In the further process of osseointegration in vivo, pronounced angiogenesis also ensures the infiltration of leukocytes [49]. In the SEM images, cells other than hOBs were occasionally identified. It can be assumed that these were introduced into the cell cultures during the isolation of the hOBs. As these were only isolated cells, it is questionable whether they could have had an influence on the course of hOB differentiation by cytokine release.

At the same time, however, the development of an inflammatory process should be prevented when inserting an endoprosthesis, as inflammation is one of the risks for inadequate anchoring of the prosthesis in the bone [50]. Using this approach, exactly the right balance must be found between an adequate immune reaction in the context of osseointegration and an excessive inflammatory reaction that impedes successful osseointegration.

### 4.2. Osseointegration Influenced by the Surface Structure

Although the surface structure of the smooth preparation turned out to be significantly smoother in the SEM than the rough surface structure, the smooth side also had a surface structure to which the cells attached and grew. Although the Rz value of approximately 10 µm can be considered a smooth structure compared to the rough surface structure, it cannot be conclusively assessed as completely smooth. The smooth side of the platelets is structured this way because of technical issues. It is normally not foreseen for direct contact with bone. The question was also raised as to whether a regular planar structure has a favourable effect on the initial attachment and adherence formation of the cells. This organised behaviour of hOBs on a regular surface has already been observed in previous studies [51]. The cells generally formed along the slight indentations but also established cell–cell contact beyond these structures (Figure 2C). In the SEM and fluorescence images, the cell density initially appeared higher on the smooth side, as the total surface area was smaller, in contrast to the rough surface structure. However, after weeks of differentiation, no qualitative difference between the rough and smooth sides could be recognised in terms of the absolute number of cells. It can be assumed that due to the initially increased cell density on the smooth side, there was less space for each individual cell, so the need for the rapid formation of elongated podocytes could be omitted. At the same time, other cells were already reached with short cell extensions, so the subsequent formation of further cell–cell contacts appeared less urgent. Morphologically, the hOBs appeared to lie planar on the preparation, which allows for the assumption that the cell–titanium contact occurred primarily via the surface. In terms of morphology, it should also be noted that the cell cytoskeleton was also planar and organised. The entire cell orientation took place along the regular surface structures, and there were only rare outbreaks beyond this [52]. It is to be determined whether, with an increasing number of cells and cell layers that are no longer in contact with the titanium surface, there is a risk that the lowest cell layer will detach. The same was also evident in the fluorescence micrographs. Here, the regular arrangement of the cell nuclei was particularly striking. It appeared as if the cell nuclei had been placed in the preformed rings. However, it remained unclear whether the cell body was then evenly arranged around the cell nucleus on the titanium.

At the same time, the rough structure turned out to be completely irregular and random, so it must be assumed that the rough structure of each tile is absolutely individual. Each platelet seemed to have a unique fingerprint. The rough side is structured in this way in order to achieve primary stability and a defined macro- and microporosity. The cells on the rough side turned out to be significantly larger than on the smooth side, and a three-dimensional formation of podocytes could be observed. After just a few days, a dense colonisation of the substrate and significantly more pronounced cell–cell and cell–titanium interactions were observed than on the smooth side. It was noticeable that there was a pronounced accumulation of cells, particularly on ‘sharp’ edges. These surrounded the cells over a large area and formed long and very thin podocytes. As required for optimal osseointegration [11], more cell–titanium contacts are created here, as the surface area is significantly larger than on the smooth side. Overall, the cell–implant contacts also appear to be much firmer. These were less widespread but more localised and in significantly more places on the titanium surface. Initially, in particular, there were significantly more cell–titanium interactions, which indicates improved adherence. Qualitatively, growth and the formation of a firm cell bond appeared to be faster on the rough side. The rough side thus appeared to be the side that offered the cells a better environment. The next question is whether there is an optimal configuration of the unevenness that enables the best possible differentiation of the cells. It is also to be determined whether a regular structure, such as that on the smooth side, initially leads to more organised growth, which could lead to more stable networks in the long term. In addition, the question arises as to whether there is an optimal rough surface configuration in terms of the distance between and the depth of the irregularities. Further investigations should follow, as no agreement has yet been reached on the optimum roughness of the implant surface [53].

It must also be determined how long the surface structure can have an influence on the formation of cell networks. Initially, a rough surface structure promotes the formation of a distinct cell network and the differentiation of the cells compared to a smooth, structured surface. After 14 d of differentiation, the rough surface structure was no longer superior, and after 21 d, comparable cell networks were formed on both surface structures (Figure 5). Only the first cell layers contact the initial surface structure; all further cell layers only contact other cells, but no longer the titanium surface. It must therefore be assumed that the surface structure can only influence the formation of cell networks for a limited time. As the first hours to days are decisive for successful osseointegration [10], it must be assumed that the rough surface structure has a clearly positive influence on this and improves it.

After just a few hours, the hOBs formed podocytes on the rough surface structure, which oriented themselves in space and not along predetermined structures. The reasons for this are still unclear [54]. One consideration is that the cells are initially more isolated and dispersed on the surface structure, and, therefore, there is a need to establish contact with other cells as quickly as possible. The question is to what extent this can be utilised. We found podocytes of several µm that extended over several levels of the network. These were very thin but firm. Further studies should be carried out to investigate how long these podocytes can become, where a limit is reached, and if the cells undergo apoptosis, for example, before they can establish cell–cell contact. The inflammatory processes that promote podocyte formation must also be investigated.

### 4.3. Influence of the Vitamins

#### 4.3.1. Vitamin K2

Both the qualitative and quantitative studies showed that the differentiation and mineralisation of the hOBs improved more under the influence of vitamin K2 than without the addition of the vitamin. In particular, the most pronounced mineralisation and differentiation were seen after 7 d and after 21 d with vitamin K2. In the qualitative studies, more pronounced podocyte formation was observed with vitamin K2. Overall, it can therefore be concluded that osseointegration is improved under vitamin K2. The possible impact of vitamin K2 on the differentiation capacity can be assumed from further studies [55]. A few previous studies have shown that coating an implant surface with vitamin K2 promotes cell growth and osseointegration [56]. When coating implants with vitamins, however, it must also be taken into account that any type of coating can also alter the adhesion behaviour of osteoblasts. Another possible application, also in a clinical context, would be the additional introduction of vitamins during the insertion of the endoprosthesis. This is closer to the design of our experiments. In addition, the oral intake of vitamin K2 is also conceivable in a clinical context to promote osseointegration. However, this would require a large number of clinical studies. 

So far, vitamin K2 has mainly appeared as a supplement to other factors [57,58]. We have now shown that the administration of vitamin K2 alone also has a significant effect on osseointegration. In particular, the effect on the degree of differentiation of the hOBs on the rough surface structure appeared significantly more pronounced than the effect of vitamin D3 (Figure 5). Ɣ-glutamyl carboxylase, for which vitamin K2 is a cofactor, appears to play a decisive role in the formation of differentiated cells and a solid ECM [59]. In addition, the synthesis of osteocalcin is dependent on vitamin K2 [60,61]. However, in the qualitative evaluation, in which calcein was used to visualise the deposited calcium complexes, no clear advantage could be seen from the administration of vitamin K2 alone. It can be assumed that vitamin K2 influences the various cells involved in bone remodelling in a variety of ways [60]. As vitamin K2 primarily intervenes in bone metabolism via inflammatory processes [62], it can be assumed that this also has a major effect on the inflammatory phase of osseointegration. Vitamin K2 also appears to play less of a role in the development of high bone density than in the development of high bone quality [63]. Both bone density and bone quality play a relevant role in successful osseointegration. The role of vitamin K2 in bone quality was supported by an observation that the number of hip fractures decreases with a high intake of the vitamin [64]. The exact mechanisms behind this have not yet been fully clarified and should be the subject of further investigations. Also, the cross-linking mechanism of vitamin K2 between its role in ECM spreading and the impact of haemostasis is not yet fully clear [65].

#### 4.3.2. Vitamin D3

In contrast to vitamin K2, vitamin D3 has long been known to have an influence on bone metabolism and osseointegration [66]. In our studies, the influence of vitamin D3 on the differentiation and mineralisation of the hOBs was lower than expected. The influence on the expression of mineralisation appeared to be similar to a slightly lower level over time than the influence of vitamin K2. In terms of differentiation, the administration of vitamin D3 alone even appeared to be inferior to the influence of vitamin K2. In the qualitative study, vitamin D3 appeared to promote mineralisation rather than the formation of cell protrusions. The influence of vitamin D3 was always less pronounced, particularly over the long course of the investigations. The influence of the concentration of the added vitamin should be discussed here. For example, it is conceivable that a change in the concentration must be made in the long-term observation, as the number or sensitivity of the vitamin D receptors decreases.

It must also be borne in mind that although the hOBs used came from non-osteoporotic patients, these individuals were not completely healthy either. They also had a reason for femoral head removal, such as a fracture event. Apart from age, gender and bone density, no further information about the patients was known to us due to the anonymisation process. It is possible that the results are affected by the age of the patients or osteoporosis with a high risk of fracture, but quite normal bone mineral density measurements. We can therefore only speculate about inter-individual characteristics. For example, it is known that obesity has an influence on the expression of vitamin D receptors [67]. Furthermore, altered vitamin D3 receptors can influence the entire immunomodulatory behaviour, so the process of osseointegration can also be influenced as a result [68]. Whether this is the case for the selected cells cannot be determined.

In vivo, vitamin D3 regulates bone mineralisation via direct and indirect effects. In our in vitro studies, we were able to investigate the direct influence of the vitamin via the vitamin D3 receptor, but not the indirect effects via the calcium and phosphate balance [69]. This may explain the weakened effects of vitamin D3. An additional dose of calcium or a coating of calcium on the titanium surface could promote the development of mineralisation under vitamin D3.

#### 4.3.3. Combination of Both Vitamins

According to the results we obtained with the individual additions of the vitamins, it was to be expected that the observed effects would complement each other. However, this could only be observed to a limited extent. While the qualitative evaluations certainly indicate that the addition of both vitamins resulted in greater podocyte formation and mineralisation, this was only confirmed quantitatively in some aspects. Initially, the combined addition significantly promoted differentiation and mineralisation. In the longer-term evaluation, there was no superiority over the addition of vitamin K2 alone, particularly in terms of the degree of differentiation. Here, too, it should be determined whether a change in the respective concentrations of the vitamin additions would have a more significant effect. Furthermore, magnesium was added to vitamin D3 and vitamin K2 in previous studies in order to optimally promote bone remodelling and the formation of the ECM [68]. In these analyses, the surfaces were also coated with vitamins and other additives; in our study, the additives were added via the differentiation medium. The idea here is that only the first cell layer that reaches the titanium surface benefits from such a coating. Due to the regular renewal of the vitamin addition, a continuous supply should be achieved that also reaches the cell layers that no longer have direct contact with the titanium surface. At the same time, however, there is the risk of cells being lifted or washed away with every change of medium. However, the qualitative images showed that the cell layers remained firm even after 21 d and numerous media changes and treatments. It must also be determined whether a change in the time of addition can lead to improved osseointegration or whether the addition of vitamin D and vitamin K2 should be delayed and not carried out together [69].

### 4.4. Reuse of the Preparations

The influence of the remaining organic residues on the rough side of the discs must also be discussed. As the organic material from previous tests could not be completely removed during cleaning, at least small amounts of residue remained. There is a well-founded suspicion that organic residues act as a growth accelerator for newly applied cells. It can therefore be assumed that a titanium surface coated with a very thin cell layer is an even better environment for hOBs. Further work needs to be performed using these approaches. 

At the same time, however, it must also be borne in mind that if organic material, such as cell residues, can remain on the surface structure, this also represents a potential risk for successful osseointegration. The rough surface structure also naturally provides a good basis for infectious agents, which can remain there if they are introduced during surgery, for example. The greatest risk for early clinical revision of an endoprosthesis is infection [70]. However, other studies have shown that the roughness of the surface structure has no influence on the adhesion of bacterial pathogens [71]. At the same time, however, it can be assumed that even if small organic residues remain on the rough surface after the most intensive mechanical and chemical cleaning, the best basis for a durable coating with a biofilm has been created. This is where research approaches come in, in which the titanium surface is coated with an antibacterial microfilm [72].

It should also be noted that there were slight signs of wear on the previously used discs. The smooth side, in particular, appeared to be affected. However, as this was not used for the qualitative analyses, it can be assumed that the reuse of the discs had at most a minor effect on the results.

## 5. Conclusions

An in vitro model was successfully established, with which we were able to show that a rough titanium surface structure leads to more adhesion points between titanium and hOBs than a smooth surface structure. In contrast, a regular and smooth surface structure leads to ordered cell networks, while an irregular surface structure also leads to more irregular but more stable networks overall. The number of cell layers and the degree of calcification were higher on the rough side. The influence of vitamin D3 on the formation of the ECM did not appear to be as great as expected. At the same time, vitamin K2 plays a more important role in osseointegration than previously assumed. The combination of both vitamins showed a clear advantage for initial adherence formation. In subsequent studies, the influence of vitamin K2 on the inflammatory phase of osseointegration must be analysed. Furthermore, the possibilities of combining the vitamins and other additives should be examined in order to increase the differentiation capacity of hOBs on titanium surfaces in the long term. It can be assumed that there is an optimal surface structure; how this should be designed and to what extent rough but also regular elements should be used must now be determined.

## Data Availability

The original contributions presented in this study are included in the article. Further inquiries can be directed to the corresponding author.

## References

[1] Ferguson R.J., Palmer A.J., Taylor A., Porter M.L., Malchau H., Glyn-Jones S. (2018). Hip replacement. Lancet.

[2] Learmonth I.D., Young C., Rorabeck C. (2007). The operation of the century: Total hip replacement. Lancet.

[3] Kurtz S., Ong K., Lau E., Mowat F., Halpern M. (2007). Projections of primary and revision hip and knee arthroplasty in the United States from 2005 to 2030. J. Bone Jt. Surg..

[4] McLaughlin J.R., Lee K.R. (2006). The outcome of total hip replacement in obese and non-obese patients at 10- to 18-years. J. Bone Jt. Surg. Br. Vol..

[5] Witte F., Kaese V., Haferkamp H., Switzer E., Meyer-Lindenberg A., Wirth C.J., Windhagen H. (2005). In vivo corrosion of four magnesium alloys and the associated bone response. Biomaterials.

[6] Guglielmotti M.B., Olmedo D.G., Cabrini R.L. (2019). Research on implants and osseointegration. Periodontology.

[7] Mavrogenis A.F., Dimitriou R., Parvizi J., Babis G.C. (2009). Biology of implant osseointegration. J. Musculoskelet. Neuronal. Interact.

[8] García-Gareta E., Coathup M.J., Blunn G.W. (2015). Osteoinduction of bone grafting materials for bone repair and regeneration. Bone.

[9] Florea D.A., Albuleț D., Grumezescu A.M., Andronescu E. (2020). Surface modification—A step forward to overcome the current challenges in orthopedic industry and to obtain an improved osseointegration and antimicrobial properties. Mater. Chem. Phys..

[10] Anselme K. (2000). Osteoblast adhesion on biomaterials. Biomaterials.

[11] Almas K., Smith S., Kutkut A. (2019). What is the Best Micro and Macro Dental Implant Topography?. Dent. Clin. N. Am..

[12] Ahmad M., McCarthy M., Gronowicz G. (1999). An in vitro model for mineralization of human osteoblast-like cells on implant materials. Biomaterials.

[13] Nobles K.P., Janorkar A.V., Williamson R.S. (2021). Surface modifications to enhance osseointegration–Resulting material properties and biological responses. J. Biomed. Mater. Res. Part B Appl. Biomater..

[14] Kim K.-H., Kwon T.-Y., Kim S.-Y., Kang I.-K., Kim S., Yang Y., Ong J.L. (2006). Preparation and Characterization of Anodized Titanium Surfaces and Their Effect on Osteoblast Responses. J. Oral Implantol..

[15] Niinomi M. (2002). Recent metallic materials for biomedical applications. Metall. Mater. Trans. A.

[16] Li J., Mutreja I., Hooper G.J., Clinch K., Lim K., Evans G., Woodfield T. (2020). Combined Infection Control and Enhanced Osteogenic Differentiation Capacity on Additive Manufactured Ti-6Al-4V are Mediated via Titania Nanotube Delivery of Novel Biofilm Inhibitors. Adv. Mater. Interfaces.

[17] Feller L., Chandran R., Khammissa R.A.G., Meyerov R., Jadwat Y., Bouckaert M., Schechter I., Lemmer J. (2014). Osseointegration: Biological events in relation to characteristics of the implant surface. S. Afr. Dent. J..

[18] Dhaliwal J.S., Marulanda J., Li J., Alebrahim S., Feine J.S., Murshed M. (2017). In vitro comparison of two titanium dental implant surface treatments: 3M^TM^ESPE^TM^ MDIs versus Ankylos^®^. Int. J. Implant. Dent..

[19] Zhao X., You L., Wang T., Zhang X., Li Z., Ding L., Li J., Xiao C., Han F., Li B. (2020). Enhanced Osseointegration of Titanium Implants by Surface Modification with Silicon-doped Titania Nanotubes. Int. J. Nanomed..

[20] Gwam C.U., Mistry J.B., Mohamed N.S., Thomas M., Bigart K.C., Mont M.A., Delanois R.E. (2017). Current Epidemiology of Revision Total Hip Arthroplasty in the United States: National Inpatient Sample 2009 to 2013. J. Arthroplast..

[21] Bozic K.J., Kurtz S.M., Lau E., Ong K., Vail T.P., Berry D.J. (2009). The Epidemiology of Revision Total Hip Arthroplasty in the United States. J. Bone Jt. Surg..

[22] Lee J.W.Y., Bance M.L. (2019). Physiology of Osseointegration. Otolaryngol. Clin. N. Am..

[23] Iwamoto J., Takeda T., Ichimura S. (2003). Treatment with vitamin D3 and/or vitamin K2 for postmenopausal osteoporosis. Keio J. Med..

[24] Franceschi R.T., James W.M., Zerlauth G. (1985). 1α, 25-Dihydroxyvitamin D _3_ specific regulation of growth, morphology, and fibronectin in a human osteosarcoma cell line. J. Cell Physiol..

[25] Datta H.K., Ng W.F., Walker J.A., Tuck S.P., Varanasi S.S. (2008). The cell biology of bone metabolism. J. Clin. Pathol..

[26] van Driel M., Koedam M., Buurman C.j., Roelse M., Weyts F., Chiba H., Van Leeuwen J.P.T.M. (2006). Evidence that both 1α,25-dihydroxyvitamin D3 and 24-hydroxylated D3 enhance human osteoblast differentiation and mineralization. J. Cell. Biochem..

[27] Christakos S., Dhawan P., Porta A., Mady L.J., Seth T. (2011). Vitamin D and intestinal calcium absorption. Mol. Cell. Endocrinol..

[28] LeBoff M.S., Greenspan S.L., Insogna K.L., Lewiecki E.M., Saag K.G., Singer A.J., Siris E.S. (2022). The clinician’s guide to prevention and treatment of osteoporosis. Osteoporos Int..

[29] Chapuy M.C., Arlot M.E., Duboeuf F., Brun J., Crouzet B., Arnaud S., Meunier P.J. (1992). Vitamin D3 and calcium to prevent hip fractures in elderly women. N. Engl. J. Med..

[30] Bouillon R., Marcocci C., Carmeliet G., Bikle D., White J.H., Dawson-Hughes B., Lips P., Munns C.F., Lazaretti-Castro M., Giustina A. (2019). Skeletal and Extraskeletal Actions of Vitamin D: Current Evidence and Outstanding Questions. Endocr. Rev..

[31] Werny J.G., Sagheb K., Diaz L., Kämmerer P.W., Al-Nawas B., Schiegnitz E. (2022). Does vitamin D have an effect on osseointegration of dental implants? A systematic review. Int. J. Implant. Dent..

[32] Olson R.E. (1984). The Function and Metabolism of Vitamin K. Annu. Rev. Nutr..

[33] Bulló M., Estruch R., Salas-Salvadó J. (2011). Dietary vitamin K intake is associated with bone quantitative ultrasound measurements but not with bone peripheral biochemical markers in elderly men and women. Bone.

[34] Boskey A.L., Gadaleta S., Gundberg C., Doty S.B., Ducy P., Karsenty G. (1998). Fourier transform infrared microspectroscopic analysis of bones of osteocalcin-deficient mice provides insight into the function of osteocalcin. Bone.

[35] Atkins G.J., Welldon K.J., Wijenayaka A.R., Bonewald L.F., Findlay D.M. (2009). Vitamin K promotes mineralization, osteoblast-to-osteocyte transition, and an anticatabolic phenotype by γ-carboxylation-dependent and -independent mechanisms. Am. J. Physiol.-Cell Physiol..

[36] Wen L., Chen J., Duan L., Li S. (2018). Vitamin K-dependent proteins involved in bone and cardiovascular health (Review). Mol. Med. Rep..

[37] Kameda T., Miyazawa K., Mori Y., Yuasa T., Shiokawa M., Nakamaru Y., Kumegawa M. (1996). Vitamin K2Inhibits Osteoclastic Bone Resorption by Inducing Osteoclast Apoptosis. Biochem. Biophys. Res. Commun..

[38] Ishida Y. (2008). Vitamin K2. Clin. Calcium.

[39] Kanis J.A. (2002). Diagnosis of osteoporosis and assessment of fracture risk. Lancet.

[40] Neidlinger-Wilke C., Stalla I., Claes L., Brand R., Hoellen I., Rübenacker S., Arand M., Kinzl L. (1995). Human osteoblasts from younger normal and osteoporotic donors show differences in proliferation and TGFβ-release in response to cyclic strain. J. Biomech..

[41] Robey P.G., Termine J.D. (1985). Human bone cells in vitro. Calcif Tissue Int..

[42] Jaiswal N., Haynesworth S.E., Caplan A.I., Bruder S.P. (1997). Osteogenic differentiation of purified, culture-expanded human mesenchymal stem cells in vitro. J. Cell Biochem..

[43] Sabokbar A., Millett P.J., Myer B., Rushton N. (1994). A rapid, quantitative assay for measuring alkaline phosphatase activity in osteoblastic cells in vitro. Bone Miner..

[44] Puchtler H., Meloan S.N., Terry M.S. (1969). On the history and mechanism of alizarin and alizarin red S stains for calcium. J. Histochem. Cytochem..

[45] Hale L.V., Ma Y.F., Santerre R.F. (2000). Semi-Quantitative Fluorescence Analysis of Calcein Binding as a Measurement of In Vitro Mineralization. Calcif. Tissue Int..

[46] Maloney W.J., James R.E., Smith R.L. (1996). Human macrophage response to retrieved titanium alloy particles in vitro. Clin. Orthop. Relat. Res..

[47] Abrahamsson I., Berglundh T., Linder E., Lang N.P., Lindhe J. (2004). Early bone formation adjacent to rough and turned endosseous implant surfaces. Clin. Oral Implant. Res..

[48] Zaporozhets T.S., Puz’ A.V., Sinebryukhov S.L., Gnedenkov S.V., Smolina T.P., Besednova N.N. (2017). Biocompatibility of Modified Osteoinductive Calcium-Phosphate Coatings of Metal Implants. Bull Exp. Biol. Med..

[49] Zetao C., Travis K., Rachael M., Ross C., Jiang C., Chengtie W., Yin X. (2016). Osteoimmunomodulation for the development of advanced bone biomaterials. Mater. Today.

[50] Anselme K., Bigerelle M., Noel B., Dufresne E., Judas D., Iost A., Hardouin P. (2000). Qualitative and quantitative study of human osteoblast adhesion on materials with various surface roughnesses. J. Biomed. Mater. Res..

[51] Lüthen F., Lange R., Becker P., Rychly J., Beck U., Nebe J.G.B. (2005). The influence of surface roughness of titanium on *β*1- and *β*3-integrin adhesion and the organization of fibronectin in human osteoblastic cells. Biomaterials.

[52] Wang B., Guo Y., Xu J., Zeng F., Ren T., Guo W. (2023). Efficacy of bone defect therapy involving various surface treatments of titanium alloy implants: An in vivo and in vitro study. Sci. Rep..

[53] Mustafa K., Wroblewski J., Hultenby K., Lopez B.S., Arvidson K. (2000). Effects of titanium surfaces blasted with TiO_2_ particles on the initial attachment of cells derived from human mandibular bone: A scanning electron microscopic and histomorphometric analysis. Clin. Oral Implant. Res..

[54] Yamaguchi M., Sugimoto E., Hachiya S. (2001). Stimulatory effect of menaquinone-7 (vitamin K2) on osteoblastic bone formation in vitro. Mol. Cell Biochem..

[55] Sarkar N., Bose S. (2020). Controlled Delivery of Curcumin and Vitamin K2 from Hydroxyapatite-Coated Titanium Implant for Enhanced in Vitro Chemoprevention, Osteogenesis, and in Vivo Osseointegration. ACS Appl. Mater Interfaces.

[56] Li H., Zhou Q., Bai B.L., Weng S.J., Wu Z.Y., Xie Z.J., Yang L. (2018). Effects of combined human parathyroid hormone (1–34) and menaquinone-4 treatment on the interface of hydroxyapatite-coated titanium implants in the femur of osteoporotic rats. J. Bone Min. Metab..

[57] Purwosunu Y., Muharram, Rachman I.A., Reksoprodjo S., Sekizawa A. (2006). Vitamin K2 treatment for postmenopausal osteoporosis in Indonesia. J. Obstet. Gynaecol. Res..

[58] Sato T., Inaba N., Yamashita T. (2020). MK-7 and Its Effects on Bone Quality and Strength. Nutrients.

[59] Myneni V.D., Mezey E. (2017). Regulation of Bone Remodeling by Vitamin K2. Oral Dis..

[60] Koshihara Y., Hoshi K. (1997). Vitamin K2 Enhances Osteocalcin Accumulation in the Extracellular Matrix of Human Osteoblasts In Vitro. J. Bone Miner. Res..

[61] Yamaguchi M., Weitzmann M.N. (2011). Vitamin K2 stimulates osteoblastogenesis and suppresses osteoclastogenesis by suppressing NF-κB activation. Int. J. Mol. Med..

[62] Sato T. Vitamin K2 and Bone Quality. Vitam Miner. https://www.omicsgroup.org/journals/Vitamin-K2-and-Bone-Quality-vms.S6-001.php?aid=21687.

[63] Yaegashi Y., Onoda T., Tanno K., Kuribayashi T., Sakata K., Orimo H. (2008). Association of hip fracture incidence and intake of calcium, magnesium, vitamin D, and vitamin K. Eur. J. Epidemiol..

[64] Maillard C., Berruyer M., Serre C.M., Dechavanne M., Delmas P.D. (1992). Protein-S, a vitamin K-dependent protein, is a bone matrix component synthesized and secreted by osteoblasts. Endocrinology.

[65] Fischer V., Haffner-Luntzer M., Amling M., Ignatius A. (2018). Calcium and vitamin D in bone fracture healing and post-traumatic bone turnover. Eur. Cell Mater..

[66] Becerra-Cervera A., Jiménez-Ortega R.F., Aparicio-Bautista D.I., López-Pérez T.V., Patiño N., Castillejos-López M., Hidalgo-Bravo A., Denova-Gutiérrez E., Salmerón J., Rivera-Paredez B. (2025). Genetic variants in vitamin D metabolism-related genes are associated with vitamin D status and adiposity markers. Nutr. Res..

[67] Christakos S., Dhawan P., Verstuyf A., Verlinden L., Carmeliet G. (2016). Vitamin D: Metabolism, Molecular Mechanism of Action, and Pleiotropic Effects. Physiol. Rev..

[68] Guler E., Baripoglu Y.E., Alenezi H., Arikan A., Babazade R., Unal S., Duruksu G., Alfares F.S., Yazir Y., Oktar F.N. (2021). Vitamin D3/vitamin K2/magnesium-loaded polylactic acid/tricalcium phosphate/polycaprolactone composite nanofibers demonstrated osteoinductive effect by increasing Runx2 *via* Wnt/β-catenin pathway. Int. J. Biol. Macromol..

[69] Gigante A., Torcianti M., Boldrini E., Manzotti S., Falcone G., Greco F., Mattioli-Belmonte M. (2008). Vitamin K and D association stimulates in vitro osteoblast differentiation of fracture site derived human mesenchymal stem cells. J. Biol. Regul. Homeost. Agents..

[70] Raphel J., Holodniy M., Goodman S.B., Heilshorn S.C. (2016). Multifunctional coatings to simultaneously promote osseointegration and prevent infection of orthopaedic implants. Biomaterials.

[71] Conserva E., Generali L., Bandieri A., Cavani F., Borghi F., Consolo U. (2018). Plaque accumulation on titanium disks with different surface treatments: An in vivo investigation. Odontology.

[72] Afewerki S., Bassous N., Harb S., Palo-Nieto C., Ruiz-Esparza G.U., Marciano F.R., Webster T.J., Furtado A.S.A., Lobo A.O. (2020). Advances in dual functional antimicrobial and osteoinductive biomaterials for orthopaedic applications. Nanomed. Nanotechnol. Biol. Med..

